# The gut microbiota of Colombians differs from that of Americans, Europeans and Asians

**DOI:** 10.1186/s12866-014-0311-6

**Published:** 2014-12-14

**Authors:** Juan S Escobar, Bernadette Klotz, Beatriz E Valdes, Gloria M Agudelo

**Affiliations:** Vidarium Nutrition, Health and Wellness Research Center, Grupo Nutresa, Calle 8 sur #50-67, Medellín, Colombia; Faculty of Engineering, University of La Sabana, Km. 7 Autopista Norte de Bogotá, Chía, Colombia; Current address: Instituto Alpina de Investigación, Alpina S.A., Km. 3 vía Briceño-Sopó, Sopó, Colombia; Current address: Faculty of Health, Institución Universitaria Colegio Mayor de Antioquia, Carrera 78 #65-46, Medellín, Colombia; Current address: Nutrition and Dietetic School, University of Antioquia, Calle 67 #53-108, Medellín, Colombia

**Keywords:** Bacterial diversity, Microbiome, Geography, Firmicutes, Bacteroidetes, Obesity, Pyrosequencing, Latin America, Colombia

## Abstract

**Background:**

The composition of the gut microbiota has recently been associated with health and disease, particularly with obesity. Some studies suggested a higher proportion of Firmicutes and a lower proportion of Bacteroidetes in obese compared to lean people; others found discordant patterns. Most studies, however, focused on Americans or Europeans, giving a limited picture of the gut microbiome. To determine the generality of previous observations and expand our knowledge of the human gut microbiota, it is important to replicate studies in overlooked populations. Thus, we describe here, for the first time, the gut microbiota of Colombian adults via the pyrosequencing of the 16S ribosomal DNA (rDNA), comparing it with results obtained in Americans, Europeans, Japanese and South Koreans, and testing the generality of previous observations concerning changes in Firmicutes and Bacteroidetes with increasing body mass index (BMI).

**Results:**

We found that the composition of the gut microbiota of Colombians was significantly different from that of Americans, Europeans and Asians. The geographic origin of the population explained more variance in the composition of this bacterial community than BMI or gender. Concerning changes in Firmicutes and Bacteroidetes with obesity, in Colombians we found a tendency in Firmicutes to diminish with increasing BMI, whereas no change was observed in Bacteroidetes. A similar result was found in Americans. A more detailed inspection of the Colombian dataset revealed that five fiber-degrading bacteria, including *Akkermansia*, *Dialister*, *Oscillospira*, Ruminococcaceae and Clostridiales, became less abundant in obese subjects.

**Conclusion:**

We contributed data from unstudied Colombians that showed that the geographic origin of the studied population had a greater impact on the composition of the gut microbiota than BMI or gender. Any strategy aiming to modulate or control obesity via manipulation of this bacterial community should consider this effect.

**Electronic supplementary material:**

The online version of this article (doi:10.1186/s12866-014-0311-6) contains supplementary material, which is available to authorized users.

## Background

Our body hosts a vast and mostly unexplored microbial world known as the human microbiota [1]. The microbiota is likely our most intimate connection with the environment. Recent investigations have highlighted the integral role these microorganisms play in human physiology, health and disease [2]. In the gastrointestinal tract, the gut microbiota is mostly composed of anaerobic bacteria of the Firmicutes and Bacteroidetes phyla [1,3,4]. These microorganisms are beneficial to the host since they confer resistance to pathogens [5], stimulate the proliferation of the gut epithelium [6], synthesize essential vitamins and regulate fat storage [7]. However, dysbiosis is associated with clinical conditions, such as obesity [8], diabetes [9,10] and cancer [11].

Regarding obesity, it has been shown that dietary changes alter the gut microbiota in a way that causes its metabolic activity to favor energy acquisition from ingested food, contribute with nutrient absorption and facilitate being stocked in adipose tissue [12,13] through a diversity of mechanisms [12,14]. It has been demonstrated that an increase caloric intake, either produced by a high-fat diet [15] or by overfeeding in genetically obese mice [16], selects an obesogenic microbiota. Studies in animal models have shown that shifts in the gut microbiota following weight gain occur in a way that causes obese animals to have proportionally less Bacteroidetes and more Firmicutes than lean animals [16-19]. In humans, however, evidence is less clear. Ley et al. [20] studied 12 obese individuals following different low-calorie diets and found that weight reduction increased the proportion of Bacteroidetes and reduced that of Firmicutes, eventually reaching the composition of lean subjects. In contrast, other authors have described modifications in the composition of the gut microbiota with weight gain in different directions [21-25].

One aspect that must be noted in the human studies is that most of them have focused on Americans or Europeans [1,26-29], giving a limited picture of the human gut microbiome. It has been established that the composition of the gut microbiota dramatically varies among individuals [1,3,30] and populations [31,32] according to the geographic [31-34] and ethnic origin [27,31,32,35], diet [15,36-39], host genetics [25,40,41], age [31,42,43] and several other factors [44-53]. An open question is how these factors interact with BMI and explain discordant results about the composition of the gut microbiota in lean and obese subjects.

To expand our knowledge of the human microbiome and determine the generality of previous observations concerning shifts in the composition of the gut microbiota following weight gain, we describe, for the first time, the gut microbiota of a group of Colombian adults using high throughput DNA sequencing and compare it with data previously obtained in other populations (USA, Europe, Japan and South Korea). Note that Colombians differ from Europeans, Americans and Asians in genetic terms, since they constitute an admixed population involving Native American, European and African ancestry in variable proportions [54-56] and have likely been exposed to different environmental conditions, including dietary habits and lifestyle [57,58]. We first asked whether the composition of the gut microbiota differs with the geographic origin of the host population. Next, we explored how BMI affects the taxonomic composition of the gut microbiota and determined whether shifts in the composition of this bacterial community following weight gain operated at broad phylogenetic scales (*e.g*., at the phylum level) or if they were produced by a reduced number of bacterial phylotypes that, eventually, might become targets to modulate or control obesity.

## Methods

We analyzed five datasets (*n* = 126): original data contributed by us from a group of 30 Colombian volunteers and four publicly available datasets from the USA, Europe, Japan and South Korea (Additional file 1: Table S1). The latter datasets were chosen because they represent comprehensive data from populations with distinct geographic origins, were directly comparable with the Colombian dataset in terms of the target population (apparently healthy adults; apparently healthy refers to the fact that no clinical examination preceded the selection process and information on health status was fully based on the self-declaration of the volunteers), used similar methods to characterize the gut microbiota (compelling diversity analyses using next-generation sequencing) and sequenced overlapping regions of the 16S gene (V2). We first describe how the new data from Colombians were obtained and then how we retrieved other data.

### Colombian dataset

We performed a cross-sectional study with apparently healthy adults of both genders from the general population living in Medellin, Colombia South America. Volunteers fulfilled the following inclusion criteria: BMI ≥18.5 kg/m^2^, were non smokers, had not been diagnosed with gastrointestinal disease, had not consumed antibiotics or antiparasitics in the last four months, had not consumed laxatives in the last two months, were not enrolled in any weight-reduction program, were not consuming weight-loss supplements, consumed less than 10 (women) or 15 (men) drinks of alcohol per week, and did not exercise for more than 10 hours per week. We enrolled 30 volunteers (16 men and 14 women) who fulfilled these criteria. Note that this sample size did not target any statistical power, since there are no previous data on Colombians and the results of studies performed on other populations are highly variable and, in many cases, contradictory [20-25]. Even among studies showing the same pattern, the magnitude of differences between lean and obese individuals is very different. Therefore, the choice of one study or another to calculate a sample size would have been totally arbitrary. Since this study constitutes a first attempt to evaluate the statistical variability of the gut microbiota among Colombians, it should be considered a pilot study. Nonetheless, its sample size is comparable to that of previous influential studies [20,59-62].

### Ethical approval

The present study was conducted according to the guidelines laid down in the Declaration of Helsinki. In addition, it was considered to have minimal risk according to the Colombian Ministry of Health (Article 11, Resolution 008430 of October 1993). All the volunteers were thoroughly informed about the study and procedures by a member of the team. Participants were assured of anonymity and confidentiality. Written informed consent was obtained from all the volunteers before beginning the study. The Institutional Ethics Committee of the University of La Sabana (Certificate 29 dated May 25, 2012) reviewed the protocol and the consent forms and approved all the procedures described here.

### Anthropometric evaluation

Weight, height and waist circumference were measured with international techniques after training and standardizing evaluators [63]. Weight was measured with a Tanita digital scale (Arlington Heights, IL; 150 kg capacity, 100 g sensibility), height with Seca mechanical measuring rods (Chino, CA; 0–200 cm range, 1 mm graduation) and waist circumference with Mabis tape measures (Waukegan, IL; 0–150 cm range, 1 mm graduation). Each measure was evaluated twice and the average of the two measures was reported. We then calculated BMI (weight in kg/height in m^2^) of participants to classify and select them according to three categories: lean (18.5 kg/m^2^ ≤ BMI < 25.0 kg/m^2^), overweight (25.0 kg/m^2^ ≤ BMI < 30.0 kg/m^2^) or obese (BMI ≥ 30.0 kg/m^2^).

### Stool sample preparation

Each participant collected a fecal sample in a hermetic, sterile recipient provided by the research team. Samples were immediately refrigerated in household freezers and brought to the laboratory within 12 hours, where a homogenized fraction was lyophilized in a Labconco 775200 Freeze Dry System (Kansas City, MO) at −50°C and 25 × 10^−3^ μbar during 48 hours, or until complete desiccation.

### DNA extraction, sequencing and taxonomic identification of bacteria

One gram of each lyophilized sample was diluted in a sterile saline solution for DNA extraction. DNA extraction was performed using the QIAamp DNA Stool Mini Kit (Qiagen; Hilden, Germany) according to the manufacturer’s instructions, using 200 μl of diluted samples. DNA was eluted from the column with 50 μl of water and diluted according to a final concentration of 20 ng/μl. DNA was quantified using a Nanodrop spectrophotometer (Nyxor Biotech; Paris, France) and sent to the Research & Testing Laboratory (Lubbock, TX) for sequencing.

DNA sequencing of the 16S rDNA was performed with the bacterial tag-encoded FLX amplicon pyrosequencing (bTEFAP) using 28F 5′TTTGATCNTGGCTCAG and 519r 5′GTNTTACNGCGGCKGCTG primers to survey the V1, V2 and V3 variable regions. Initial generation of the sequencing library utilized a one-step PCR with a total of 30 cycles, a mixture of Hot Start and Hot Star high fidelity *Taq* polymerases, and amplicons originating and extending from the 28F primer for bacterial diversity. The bTEFAP utilized the Roche 454 FLX instrument with titanium reagents and titanium procedures. The average sequencing depth was 10 K reads per assay.

Following DNA sequencing, all failed sequence reads (*i.e*., those not passing any of the filters considered in the Roche 454 signal processing pipeline, (available at http://454.com/downloads/my454/documentation/gs-junior/software-manual/454_Sequencing_Software_Manual_v2.5p1_PartB.pdf); briefly, the signal processing performs a series of normalization, correction and quality filtering steps and outputs the remaining [high quality] signals into flowgrams for each read), low quality sequence ends (Q < 15), barcodes and primers were removed, and sequence collections depleted of any non-bacterial rDNA sequence and chimeras using B2C2 [64]. To determine the identity of bacteria in the remaining reads, DNA sequences were filtered (minimum sequence length = 150 bp; maximum sequence length = 1000 bp; number of ambiguous bases < 6; mean quality score > 25; no mismatches were allowed in primers), assigned to samples based on their nucleotide barcode, assembled into clusters of operational taxonomic units (OTUs) based on their sequence similarity using uclust [65] and PyNAST [66], and queried against the Greengenes database [67], 12_10 release, using the RDP classifier [68] implemented in QIIME 1.5.0-dev [69]. Sequence identity ≥80%, ≥95% and ≥97% delimited taxonomy at the phylum, genus and species levels, respectively. Although determining exactly how OTUs should be defined is an active area of research [70,71], we adhered to these commonly used values for the sake of comparability with previous studies [31,62,72,73]. Phylogenetic trees and OTU tables were constructed for each dataset with QIIME. The analysis pipeline is provided as Additional file 1: Figure S1. Raw sequences were deposited at the European Nucleotide Archive [EMBL: ERP003466]. Assembled sequences are available as Additional file 2.

### American, European and Asian datasets

We retrieved and analyzed 16S rDNA sequences from some previous studies: USA [41], Europe [27], South Korea [62] and Japan [72]. Although in all these studies the BMI of volunteers was recorded, in the USA and European datasets only lean, overweight and obese volunteers were recruited; the Japanese and Korean datasets focused almost exclusively on lean individuals.

**Figure 1:**
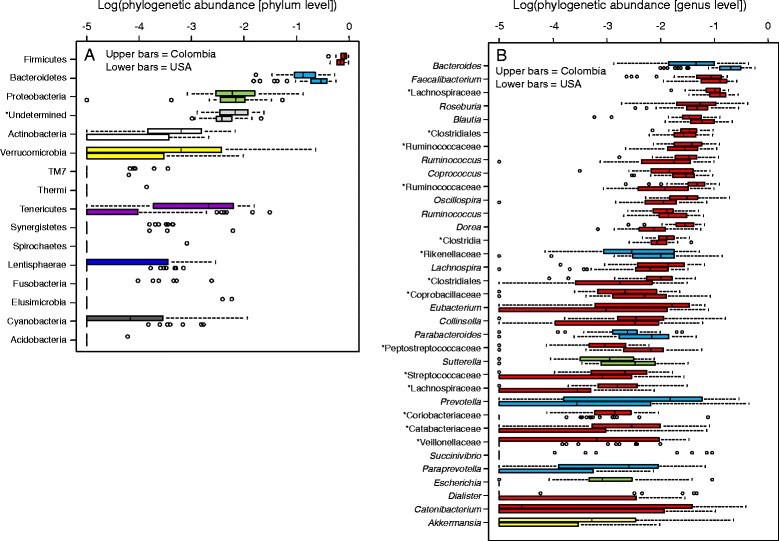
**Taxonomic profiles of the gut microbiota of Colombians and Americans. (A)** Relative abundance of phylum-level OTUs. **(B)** Relative abundance of the most frequent genus-level OTUs (frequency >0.5%), colored by their respective phylum (see Figure A). Unclassified phylotypes are marked with asterisk. Upper bars = Colombians; lower bars = Americans.

For the USA dataset, where the gut microbiota of obese and lean female twins and their mothers was characterized [41], we downloaded final 454-generated V2 16S rDNA sequences (available at http://gordonlab.wustl.edu/SuppData.html) and extracted reads from 54 twins (the first coded twin of each twin pair). We refrained from analyzing the two twins or the twin-mother couple because relatedness is a source of within-population community similarity (see Figure 1A in reference [41]) that might exacerbate statistical differences among populations. In addition, by restricting analyses to unrelated individuals we made all datasets directly comparable. Also, for the sake of comparability, we only analyzed the first fecal sample (out of two) of each subject.

The European dataset consisted of the subset of 13 healthy volunteers (three women and 10 men) among Spanish, French and Danish inhabitants whose microbiomes were published by the MetaHIT Consortium [27]. For the sake of comparability with the other studies, Italians were not analyzed because they were elders. Raw sequences were downloaded from the NCBI Trace Archive (see Additional file 1: Table S1 for accession numbers) and complete 16S gene fragments extracted using BLASTN searches against the Greengenes 12_10 database (e-value <10^−5^; bit-score >50; %identity >50; alignment length ≥100).

The Korean dataset consisted of 14 lean and four overweight individuals (six women and 12 men) in which the V1, V2 and V3 16S rDNA regions were sequenced [62]. Originally denoised and filtered 454-generated sequences were kindly provided by Dr. Young-Do Nam. For comparability with the other studies, we analyzed only the first stool sample (out of three) of individuals A–F (*i.e*., A0–F0), and the only stool sample of individuals I–T. For comparability with the other datasets, we did not analyze individuals G and H since they were children six and four years old, respectively.

Finally, the Japanese dataset consisted of 454-generated V1 and V2 16S rDNA sequences of 10 lean and one overweight adults (six females and five males) that participated in an intervention with probiotics [72], available at the NCBI’s SRA database (queried on October 21, 2013; see Additional file 1: Table S1 for accession numbers). For comparability with the other datasets, we only analyzed sequences obtained before the probiotic intervention.

To compare datasets, we extracted the only common 16S rDNA region to the five studies (*i.e*., the V2 region) using the V-Xtractor 2.1 [74]. V2 sequences were assembled into OTUs, aligned and queried against Greengenes 12_10 using the same procedures described above (Additional file 1: Figure S1).

### Statistical analysis

The gut microbiota of each individual in each dataset was first summarized by taxonomic composition to obtain α-diversity estimates. Rarefaction curves were constructed using Chao-1, the number of species-level OTUs and phylogenetic distance using QIIME. We then assessed the β-diversity of the gut microbiota using multivariate UniFrac analyses. UniFrac measures differences between microbial communities based on phylogenetic information; its premise is that two microbial communities with a shared evolutionary history share branches on a phylogenetic tree and that the fraction of branch length shared can be quantified and interpreted as the degree of community similarity. We restricted analyses to unweighted UniFrac distances because heterogeneity in sequencing depth between studies. Unweighted distances consider only changes in species composition (*i.e*., presence–absence) [75]. UniFrac distances were obtained with Fast UniFrac [76] using rarefied data (depth = 100 sequences/sample). Comparisons among populations (Colombia, USA, Europe, Japan and Korea), BMI categories (lean, overweight and obese) and gender (male and female) utilized the analysis of similarity (ANOSIM) and the *adonis* function for permutational multivariate analysis of variance implemented in QIIME.

Next, we tested hypotheses put forward in previous studies concerning shifts in the taxonomic composition of the gut microbiota between lean and obese subjects in more detail. For this, we performed linear regressions on the proportions (bacterial taxon/total bacteria) of phylum-level OTUs using population, BMI, age and gender as independent variables. In addition, since it has recently been suggested that latitude would be the main underlying factor explaining between-population differences in Firmicutes and Bacteroidetes [34], we correlated latitude with the proportions of these two phyla using Pearson’s *r*. When comparing populations, analyses were performed on bacterial proportions because total bacterial counts were significantly different among datasets (*F*_4, 108_ = 147.02, *P* < 0.0001).

Since the Colombian, USA and European datasets contained lean, overweight and obese individuals, we analyzed them separately to test the effect of BMI on the composition of the gut microbiota in each population independently. In these cases, we analyzed the proportions as well as the counts of phylum-level OTUs and controlled for possible confounding factors (gender, age and waist circumference in the Colombian dataset; ancestry [European or African] and age in the USA dataset; country of origin [Spain, France or Denmark], gender and age in the European dataset). In addition, we performed univariate *F*-tests and correlation analysis (Pearson’s *r*) in these three datasets to investigate the correlations between genus-level OTUs and BMI. Where necessary, *P*-values were adjusted for multiple comparisons [77].

In all analyses, bacterial counts were log-transformed and proportions were arcsin-square-root transformed to guarantee the normal distribution of residuals and homoscedasticity, tested using the Shapiro-Wilk and Fligner-Killeen tests, respectively. Note that in genus-level analyses, some individuals had no bacterium of a given genus (*i.e*., a count of zero sequences for that OTU) and logarithmic transformation was impossible. However, these data were important because they represented extreme values. Rather than removing them, in these analyses we used the transformation log(1 + *x*_*i*_). General statistical analyses were performed with R 2.15.2 [78].

## Results

Some characteristics of the different datasets are shown in Table 1. This table indicated that individuals with excess weight tended to be older than lean individuals; although the tendency was not significant, except in the Japanese dataset, it justified controlling for age in statistical models. Table 1 also showed that, in the Colombian dataset, waist circumference increased significantly at higher BMI, indicating central obesity and justifying taking this variable into account in analyses.

**Table 1:** **General characteristics of the different datasets**

**Dataset** – **Variable**	**Lean**	**Overweight**	**Obese**	***P***
*Colombia*				
Age (years)	33 ± 11	39 ± 9	43 ± 12	0.10
Weight (kg)	62.2 ± 8.0	73.5 ± 7.2	90.1 ± 8.0	<0.0001
Height (m)	1.655 ± 0.085	1.647 ± 0.070	1.663 ± 0.056	0.88
BMI (kg/m^2^)	22.6 ± 1.7	27.1 ± 1.3	32.6 ± 2.3	<0.0001
WC (cm)	78.5 ± 6.4	91.9 ± 7.4	107.8 ± 8.2	<0.0001
*Europe*				
Age (years)	56 ± 9	56 ± 9	59 ± 6	0.78
BMI (kg/m^2^)	22.5 ± 1.2	28.4 ± 0.8	32.8 ± 1.7	<0.0001
*Japan*				
Age (years)	21 ± 1	33	NA	<0.0001
BMI (kg/m^2^)	20.3 ± 0.8	28.0	NA	<0.0001
*South Korea*				
Age (years)	43 ± 16	58 ± 13	NA	0.09
BMI (kg/m^2^)	22.5 ± 1.2	28.5 ± 0.6	NA	<0.0001
*USA*				
Age (years)	26 ± 2	26 ± 3	26 ± 3	0.73
BMI (kg/m^2^)	21.3 ± 1.0	28.3 ± 0.6	41.7 ± 7.8	<0.0001

Data presented as average ± standard deviation; *P*-values from ANOVA testing differences among lean, overweight and obese subjects. *WC* = waist circumference; *NA* = not available.

### Geographic variability of the gut microbiota

In the new dataset contributed here on Colombians, we obtained 509,147 16S rDNA sequences from the stool samples of the 30 volunteers. Of these, 466,010 sequences passed QIIME quality filters and were subsequently analyzed. The minimum/average/maximum sequence counts per individual were 10,229/15,534/21,825, respectively, and the minimum/average/maximum sequence length was 173/318/529 bp, respectively. These sequences clustered into 16,810 different species-level OTUs (*i.e*., sequences differing >3%), of which 15,866 could be assigned a phylum name and 5864 a genus name. Note that the remaining sequences were correctly clustered by percentage of identity but were assigned a higher taxonomic rank.

The comparison between the numbers of observed species-level OTUs and the Chao-1 estimator in the Colombian dataset suggested that, at the depth of our sequencing, we sampled about half the bacterial diversity hosted in the gut of these volunteers. Additional sampling would be needed to capture the remaining diversity, made of species present at very low abundance (<0.005% of an individual’s gut bacterial diversity). The tendency was rather similar in the other datasets (Additional file 1: Figure S2). This is a common limitation to most bacterial diversity studies and indicates that rare components of the gut microbiota are difficult to detect at the depth of common sequencing. However, even if rare species make an important contribution to the total gut diversity, dominant species (*i.e*., those contributing the most to the ecosystem biomass) are expected to be the main determinants of ecosystem processes [79]. It is, therefore, reasonable to focus on dominant species to investigate differences between populations and test previous observations concerning shifts in the gut microbiota following weight gain.

We found that the gut microbiota of Colombians was mostly composed of Firmicutes (average ± SD: 79 ± 13%) and Bacteroidetes (17 ± 12%), followed by other phyla present in minor frequencies (Figure 1A). However, variation among volunteers in the proportion of these phyla was notorious, with some individuals having up to 97% of their gut microbiota composed of Firmicutes and less than 2% of Bacteroidetes, and others having 40% of Firmicutes and 53% of Bacteroidetes (Additional file 1: Figure S3). The remaining datasets had lower proportions of Firmicutes and higher proportions of Bacteroidetes (Table 2), but dispersion of data among individuals was equally notorious than in the Colombian dataset (results not shown). In the Japanese, there was a higher proportion of Actinobacteria than in the other datasets (Table 2).

**Table 2:** **Taxonomic composition of the gut microbiota in the different datasets**

**Phylum**	**Colombia**	**Europe**	**Japan**	**South Korea**	**USA**	***P***
Actinobacteria	0.001 ± 0.002	0.008 ± 0.023	0.182 ± 0.238	0.000 ± 0.000	0.000 ± 0.000	<0.0001
Bacteroidetes	0.166 ± 0.119	0.306 ± 0.161	0.179 ± 0.171	0.262 ± 0.180	0.287 ± 0.141	0.005
Firmicutes	0.787 ± 0.128	0.589 ± 0.173	0.626 ± 0.211	0.689 ± 0.213	0.696 ± 0.144	0.012
Proteobacteria	0.020 ± 0.033	0.013 ± 0.011	0.012 ± 0.013	0.015 ± 0.012	0.010 ± 0.010	0.10
Tenericutes	0.004 ± 0.005	0.016 ± 0.049	0.000 ± 0.000	0.006 ± 0.008	0.001 ± 0.005	0.0007
Verrucomicrobia	0.012 ± 0.042	0.012 ± 0.026	0.000 ± 0.001	0.000 ± 0.000	0.001 ± 0.002	<0.0001

Data presented as average ± standard deviation; *P*-values from ANOVA testing differences among lean, overweight and obese subjects. *WC* = waist circumference; *NA* = not available.

**Figure 2:**
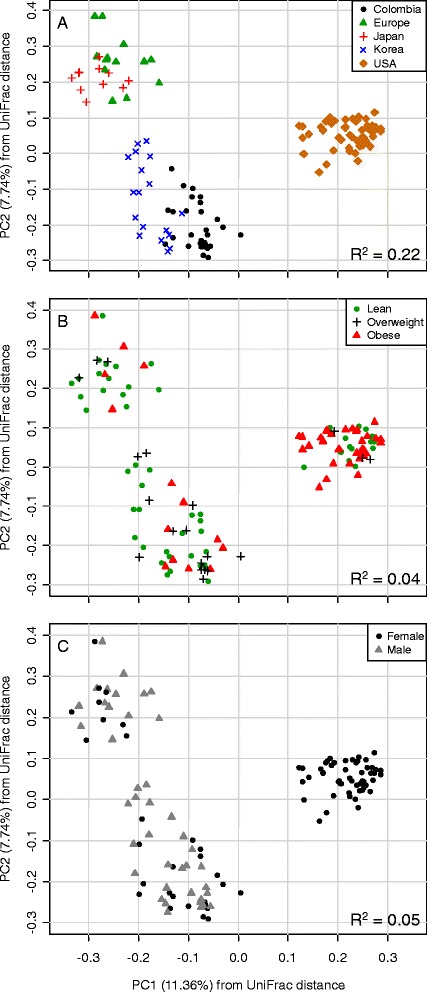
**Principal correspondence analysis of UniFrac distances.** Differences in the composition of the gut microbiota according to the geographic origin of the sampled population **(A)**, nutritional status **(B)** and gender **(C)**. R^2^ and *P*-*value* from permutational multivariate analysis of variance (*adonis* function).

The UniFrac analysis indicated that the gut microbiota of Colombians was significantly different from that of Americans, Europeans and Asians (*adonis*: R^2^ = 0.22, *P* = 0.001; ANOSIM: R = 0.78, *P* = 0.001). Indeed, the geographic origin of the population was the most relevant grouping factor in the analysis of the gut microbiota of Colombians, Americans, Europeans, Japanese and Koreans, above BMI (*adonis*: R^2^ = 0.04, *P* = 0.001; ANOSIM: R = 0.11, *P* = 0.002) or gender (*adonis*: R^2^ = 0.05, *P* = 0.001; ANOSIM: R = 0.26, *P* = 0.001) (Figure 2). On the other hand, our results did not support the hypothesis that Firmicutes increases and Bacteroidetes decreases with latitude [34]. In contrast, in the five datasets analyzed here, we found that the relative abundance of Firmicutes decreased with latitude (*r* = −0.27, *P* = 0.002) and that of Bacteroidetes increased with latitude (*r* = 0.28, *P* = 0.001) (Additional file 1: Figure S4).

### Composition of the gut microbiota following weight gain

**Figure 3:**
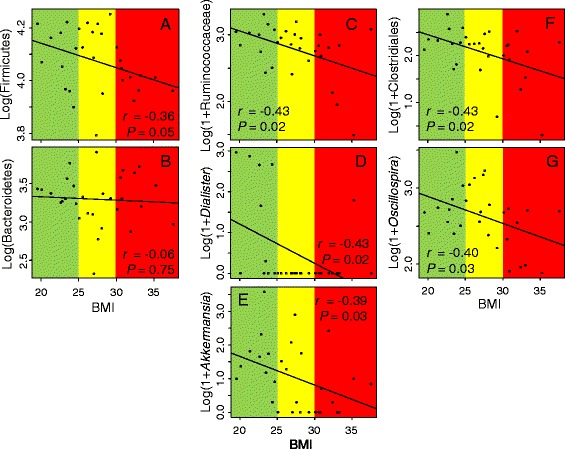
**Changes in the abundance of phylum-level and genus-level OTUs with BMI in the Colombian dataset. A**-**B**: phylum-level OTUs; **C**-**G**: genus-level OTUs. Background color: green = lean; yellow = overweight; red = obese. Pearson’s *r* from correlation analyses and *P*-*value* from linear models.

We found that Firmicutes tended to be less abundant at a higher BMI in the Colombian dataset when controlling for gender, age and waist circumference (*F*_1, 25_ = 4.04, *P* = 0.05, *r* = −0.36). No change was observed for Bacteroidetes though (*F*_1, 25_ = 0.10, *P* = 0.75, *r* = −0.06) (Figure 3A-B). A similar result was found in the USA dataset (Firmicutes: *F*_1, 50_ = 5.68, *P* = 0.02, *r* = −0.30; Bacteroidetes: *F*_1, 50_ = 0.58, *P* = 0.45, *r* = −0.23). In the European dataset there was no change in Firmicutes or Bacteroidetes with BMI (Firmicutes: *F*_1, 7_ = 0.93, *P* = 0.37, *r* = 0.25; Bacteroidetes: *F*_1, 7_ = 0.005, *P* = 0.95, *r* = −0.08).

We then looked in more detail to see which of the most representative phylotypes, binned at 95% sequence identity (*i.e*., genus-level OTUs), changed their abundance with an increasing BMI. In the Colombian dataset, 200 different genus-level OTUs were identified; 30 of them occurred at frequencies greater than 0.5% and, together, represented 91.4% of the total diversity of the gut bacteria (23 Firmicutes, four Bacteroidetes, two Proteobacteria and one Verrucomicrobia). In this dataset, an undetermined Lachnospiraceae, *Faecalibacterium* and *Roseburia* were predominant among Firmicutes, whereas *Bacteroides* and *Prevotella* were the most abundant Bacteroidetes (Figure 1B). We detected that five out of the 30 most abundant genus-level phylotypes present in this dataset suffered reductions with an increasing BMI: four Firmicutes (Ruminococcaceae, Clostridiales, *Dialister* and *Oscillospira*) and one Verrucomicrobia (*Akkermansia*) (Figure 3C-G). The other datasets had lower species richness but similar numbers of the most prevalent phylotypes than the Colombian dataset. In the USA dataset, among the most prevalent genera *Bacteroides*, *Coprococcus*, *Oscillospira*, *Parabacteroides*, undetermined Clostridia, Clostridiales, Rikenellaceae and Ruminococcaceae diminished with BMI whereas *Catenibacterium* became more abundant at a higher BMI. In Europeans, *Bacteroides* became less abundant and undetermined Veillonellaceae, which was more abundant at a higher BMI (Additional file 3: Table S2).

## Discussion

### Geographic variation of the gut microbiota

The gut microbiota is currently recognized as an organ that interacts in a complex way with the human body. These bacteria play a fundamental role both in maintaining gut health and contributing to several pathologies [80-83]. Recent research has emphasized the relationship between bacterial composition and obesity [16,17,20,41,84]. However, there is no consensus about what the “typical” gut microbiota of obese and lean subjects would be. One of the reasons for this is that we have a limited understanding of the extent to which this relationship is affected by factors such as the geographic origin of the surveyed population. Most studies in humans have focused on Americans or Europeans [1,20-22,24,41,59,61,85-87] and few have done tests in populations with different geographic and genetic origins [23,27,32,35]. Yet, it has been established that genetic background and geography are some of the most important determinants of the gut bacterial composition [25,31,33,34,40,41]. For instance, a study comparing the gut microbiota of subjects from the Amazonas of Venezuela, rural Malawi and USA metropolitan areas found that the origin of the population primarily explains the variation in the composition of this bacterial community [31]. Likewise, another recent study, in which the gut microbiota of Hazdas, Burkinabes, Malawians, Italians and Americans was compared, found that geography was clearly the most important grouping factor [32]. In agreement with this, we here show, using new data in overlooked Colombians, that the origin of the population explains more variability in the composition of the gut microbiota than factors such as BMI or gender.

A recent study suggested a link between latitude and the prevalence of Firmicutes and Bacteroidetes in a sort of Bergman’s rule, where populations living in higher latitudes tend to have a larger body mass and relatively more Firmicutes and less Bacteroidetes than in populations at lower latitudes [34]. In contrast with such a hypothesis, our results on Colombians suggest that individuals from this population have a higher proportion of Firmicutes and a lower proportion of Bacteroidetes than expected according to Colombia’s latitude.

An interesting result obtained with the UniFrac analysis was that the taxonomic composition of the gut microbiota of Colombians and Koreans, and Europeans and Japanese were partially overlapping. Whereas it is hard to impute such resemblance to host genetic similarities, it is tempting to assign it to shared environmental factors, such as macronutrient intake. According to national health and nutrition surveys, the energy intake of Colombians (average of males and females 19–50 years old = 1869 Kcal/day) [57] is closer to that of Koreans (average of males and females in 2007 = 1806 Kcal/day) [88] than to intakes of Japanese (average of males and females 20–49 years old = 1945 Kcal/day) [89], Americans (average of males and females 20–49 years old = 2278 Kcal/day) [90] or Europeans (average of French, Spanish and Danish 19–64 years old = 2281 Kcal/day) [91]. Such lower energy intake in Colombians and Koreans is due to an average diet lower in total fat (Korea = 37.1 g/day, Colombia = 49.0 g/day, Japan = 59.3 g/day, USA = 82.9 g/day, Europe = 95.3 g/day), lower protein content (Colombia = 59.0 g/day, Korea = 66.4 g/day, Japan = 69.6 g/day, USA = 87.3 g/day, Europe: = 98.9 g/day) and higher carbohydrate intake (Europe = 229.8 g/day, Japan = 263.6 g/day, USA = 279.5 g/day, Colombia = 290.7 g/day, Korea = 301.7 g/day). Likewise, fiber intake seems to be higher in Koreans (19.8 g/day) [92] and Colombians (18.2 g/day) [57] than Americans (15.1 g/day) or Japanese (15.0 g/day) [62]. Although this is mere speculation and we do not pretend to claim causality with such rough values, it would be interesting to tease apart the effect of diet and geography on the composition of the gut microbiota.

### Composition of the gut microbiota in lean and obese individuals

Several authors have given support to the observation that Firmicutes increases and Bacteroidetes decreases in obese compared to lean subjects [17,20]. In one of the most influential studies to date analyzing the gut microbiota of 154 individuals (mothers + twins) with different BMI by means of 454 pyrosequencing of the V2, V6, complete 16S and whole metagenome, Turnbaugh et al. [41] found less Bacteroidetes in obese subjects than in those who were lean. However, they did not detect any difference among Firmicutes. The difference between the original publication and our results in the USA dataset (*i.e*., a fraction of the original data where Firmicutes diminished with BMI and Bacteroidetes did not change) is likely that the two studies performed different analyses (comparison between relative abundance of bacteria between lean and obese in the former; correlation between bacterial counts and BMI in the latter) and that Turnbaugh et al. [41] analyzed much more data than we did, which gave them greater statistical power. Using a smaller sample (49 individuals) and a different bacterial identification technique (quantitative PCR), Armougom et al. [85] found the same results of Turnbaugh et al. [41]. In contrast with these studies, other authors have described shifts in the gut microbiota with BMI in the opposite sense: a higher proportion of Bacteroidetes [21,24] and a lower proportion of Firmicutes [22,24,25] in individuals with excess weight compared to lean subjects. Furthermore, other studies have detected increases in both phyla [23] or, more commonly, no difference in their abundance with increasing BMI [1,27,35,59,60,87]. Our results indicated that, similar to previous studies [1,3,62], Firmicutes and Bacteroidetes were the dominant bacterial phyla colonizing the gut of Colombians. These two phyla constituted >95% of the phylotypes detected in this dataset. Nonetheless, differences in their abundance between individuals, which also occur in the other datasets analyzed here, suggest that there are complex genotype-by-environment interactions that contribute to maintain the bacterial community structure in the face of immune, environmental and lifestyle/dietary exposures. The uniqueness of each individual’s microbial community is a universal feature of the human microbiome [1,3,41]. However, results in the Colombian dataset did not agree with the observation of increased Firmicutes and reduced Bacteroidetes in individuals with a higher BMI. We found less Firmicutes in volunteers with a higher BMI, as observed by others [22,24,25], and no shift in Bacteroidetes [22,59].

Contradictory results between studies on obesity and phylum-level changes on the gut microbiota are common and have deserved explanations. Inspection of studies revealed they are heterogeneous in several aspects. Whereas some of them, including the new data contributed by us in Colombians, assessed bacterial diversity using broad rDNA surveys and high throughput sequencing [1,27,31,41,60,87], others performed analyses based on taxon-specific oligonucleotide probes [21,22,24,25,35,59,61,85,86]. The latter techniques are limited by the specificity of the selected probes, which is uncertain in the absence of large rDNA surveys that assess the overall diversity within a sample [8]. Another methodological issue that affects comparability between studies is the use of different taxonomic databases to classify 16S rDNA sequences. Our choice of Greengenes was based on the fact that this is a curated, quality-checked database with millions of sequences that has been proved to improve the classification of 75% of the sequences by one or more taxonomic ranks related to the NCBI [93]. Sample size is another issue that can contribute to disagreement among studies. While some of them analyzed as few as nine or 12 individuals [20,60] others sampled 100 subjects or more [24,31,35,41,87]. A higher sample size reduces sampling stochasticity and increases statistical power. Other factors, such as the duration of the fasting period at the moment of sampling [8] or the storage conditions of stool samples prior to DNA extraction [94], could also contribute to differences among studies.

However, as suggested above, a more fundamental aspect that profoundly affects comparability among studies is the geographic origin of the sampled population. Populations differ in two domains: genetic (*i.e*., the genetic background itself as well as the genetic variants involved in susceptibility to metabolic disorders, inflammation and host-bacteria symbiosis) and environmental (*e.g*., diet content, lifestyle). Studies in laboratories with animal models usually lack genetic variation and control macro-environmental variables, which might explain why results in obese and lean animals are more consistent than in humans [15-19,95-97]. Since in human studies such controls are not possible, it is important to split apart the contributions of geography and BMI (and other factors) to changes in this bacterial community.

Although pioneering studies associated obesity with phylum-level changes in the gut microbiota, studies finding correlations at lower taxonomic levels are becoming more abundant. Ley et al. [17] did not find differences in any particular subgroup of Firmicutes or Bacteroidetes with obesity, which made them speculate that factors driving shifts in the gut microbiota composition must operate on highly conserved traits shared by a variety of bacteria within these phyla [20]. However, more recent evidence suggested that specific bacteria might play determinant roles in the maintenance of normal weight [98], in the development of obesity [99] or in disease [80,100-103]. In this study, we found that a reduced set of genus-level phylotypes was responsible for the reductions at the phylum level with an increasing BMI. In Colombians, the phylotypes that became less abundant in obese subjects were related to degradation of complex carbohydrates [25,27,104] and had been found to correlate with normal weight [25,60,86,98,105-107]. Results in this population suggest that a lower BMI associates with the presence of primary-fiber degraders and that these bacteria impact the energy balance of the host. They might represent promising avenues to modulate or control obesity in this population.

## Conclusion

Studies examining the gut microbiota outside the USA and Europe are beginning to be accumulated. They expand our knowledge of the human microbiome. This study contributed to this aim by describing, for the first time, the gut microbiota of unstudied Colombians. We showed that the geographic origin of the studied population was a more important factor driving the taxonomic composition of the gut microbiota than BMI or gender. Strategies to modulate or control obesity via intervention of the gut microbiota should take this effect into account.

### Availability of supporting data

Raw sequences of Colombians supporting the results of this article are available in the European Nucleotide Archive [EMBL: ERP003466], of Europeans in the NCBI Trace Archive [Trace Archive: 33049, 33053, 33055, 33057, 33061, 33063, 33305, 33307, 33309, 33313, 38231, 38233, 45929], and of Americans, Japanese and Koreans in the NCBI Short Read Archive [Americans: SRX001342, SRX001345, SRX001348, SRX001351, SRX001354, SRX001357, SRX001445, SRX001447; Japanese: DRX002796, DRX002805, DRX002814, DRX002823, DRX002832, DRX002841, DRX002850, DRX002859, DRX002867, DRX002875, DRX002884; Koreans: DRX000481].

## Additional files

Additional file 1: Table S1. Some characteristics of the different datasets analyzed in this study. **Figure S1** - Analysis pipeline. **Figure S2** - Rarefaction curves in the different datasets. **Figure S3** - Inter-individual variability of the gut microbiota among Colombians. **Figure S4** - Correlations between the relative abundance of Firmicutes and Bacteroidetes with latitude. Additional file 2:
**Assembled sequences of the Colombian dataset (in Fasta format).**
Additional file 3:
**Correlation analyses between genus-level OTU abundance and BMI for the Colombian, American and European datasets.**

## Abbreviations

ANOSIM: Analysis of similarity
BMI: Body mass index
bTEFAP: bacterial tag-encoded FLX amplicon pyrosequencing
OTU: Operational taxonomic unit
rDNA: ribosomal DNA
