# Supplementary material for: The gut microbiota of Colombians differs from that of Americans, Europeans and Asians
Source: BMC Microbiol. 2014 Dec 14;14:311. doi: 10.1186/s12866-014-0311-6 (PMC4275940; doi:10.1186/s12866-014-0311-6)

Supplementary Materials

Supplementary Table S1 - Some characteristics of the different datasets analyzed in this study

Population	n	16S region	BMI (kg/m^2)	Gender (M: F)	Age (years)	Database: accession numbers	Reference
**Colombia**	30	V1–V3	19.5–37.6	16: 14	21–60	EMBL: ERP003466	This study
**Europe**	13	Full length	20.5–35.2	10: 3	38–64	NCBI Trace Archive: MH6 (33049), MH13 (33053), MH12 (33055), MH30 (33057), CD2 (33061), UC6 (33063), NO1 (33305), NO3 (33307), NO4 (33309), OB2 (33313), OB1 (38231), OB6 (38233), OB8 (45929)	[27]
**Japan**	11	V1–V2	19.4–28.0	5: 6	19–33	SRA: DRX002796, DRX002805, DRX002814, DRX002823, DRX002832, DRX002841, DRX002850, DRX002859, DRX002867, DRX002875, DRX002884	[72]
**South Korea**	18	V1–V3	21.2–29.2	12: 6	27–68	SRA: DRX000481	[62]
**USA**	54	V2	19.0–64.0	0: 54	21–31	SRA: SRX001342, SRX001345, SRX001348, SRX001351, SRX001354, SRX001357, SRX001445, SRX001447	[41]

Supplementary Figure S1 - Analysis pipeline

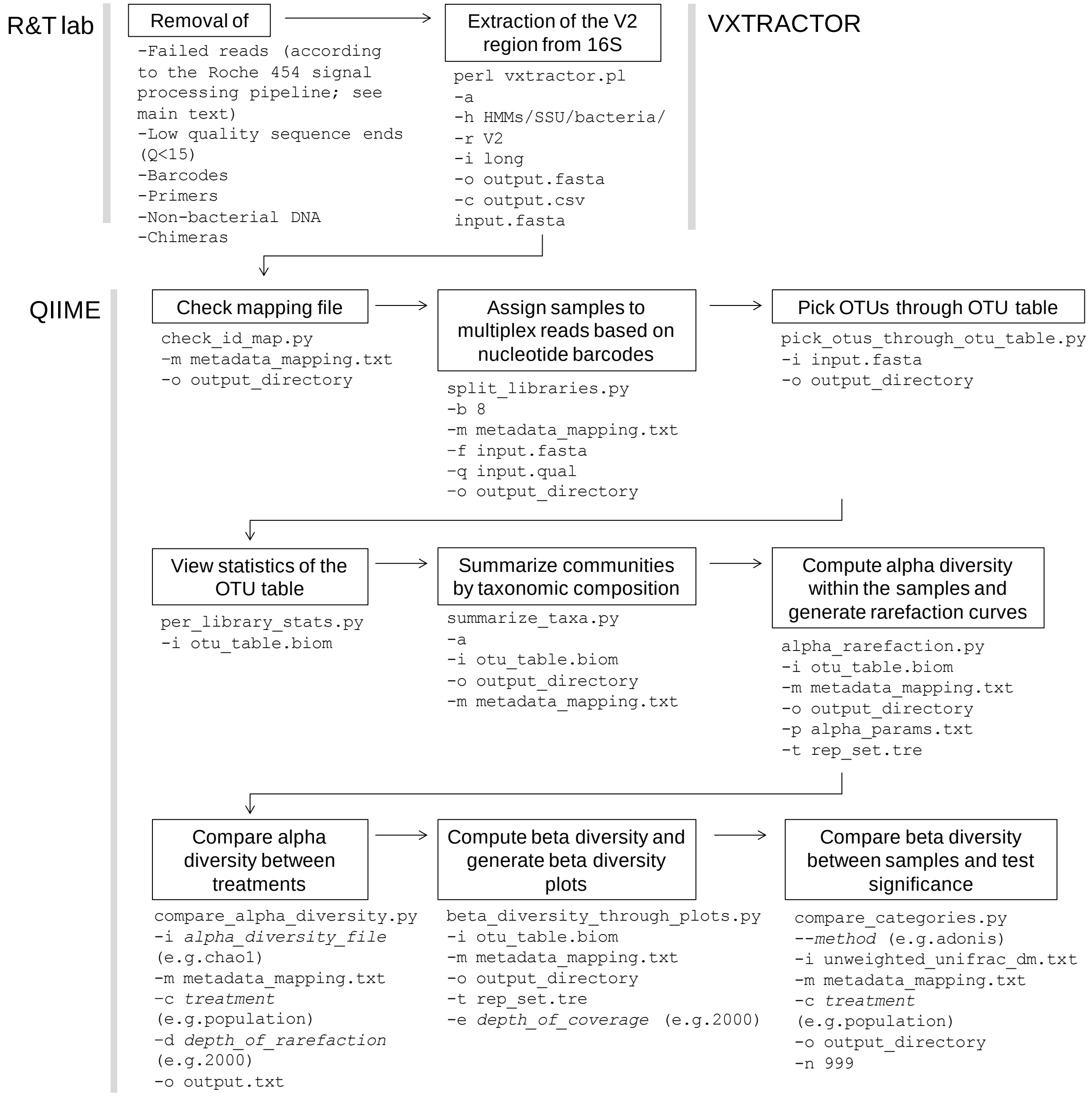

Supplementary Figure S2 - Rarefaction curves in the different datasets

Bacterial diversity in the gut microbiota of the different datasets according to the Chao-1 estimator (left) and the number of observed species-level OTUs (right). Green = lean; yellow = overweight; red = obese.

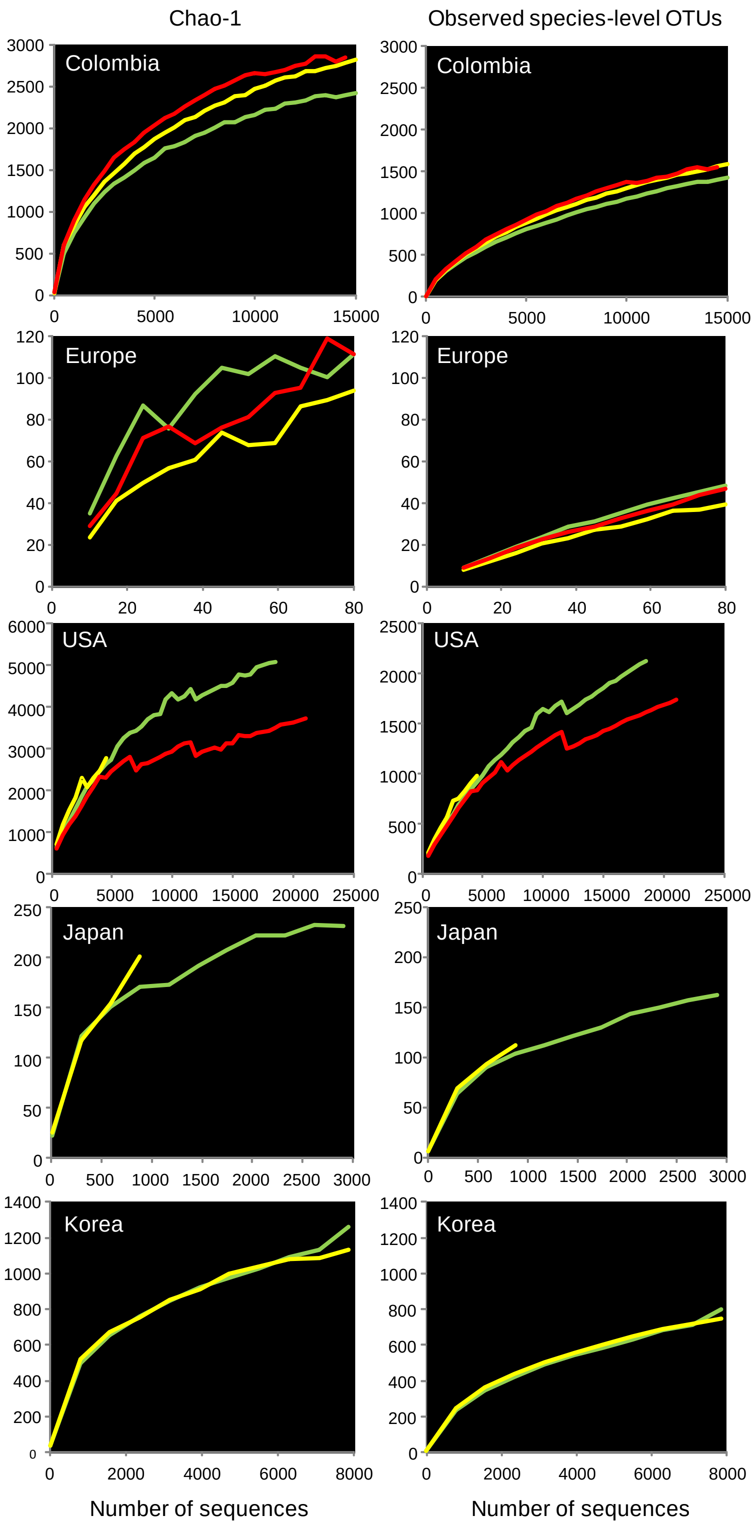

Supplementary Figure S3 - Inter-individual variability of the gut microbiota among Colombians

Relative contribution of phylum-level OTUs to the gut microbiota of Colombian adults according to their nutritional status.

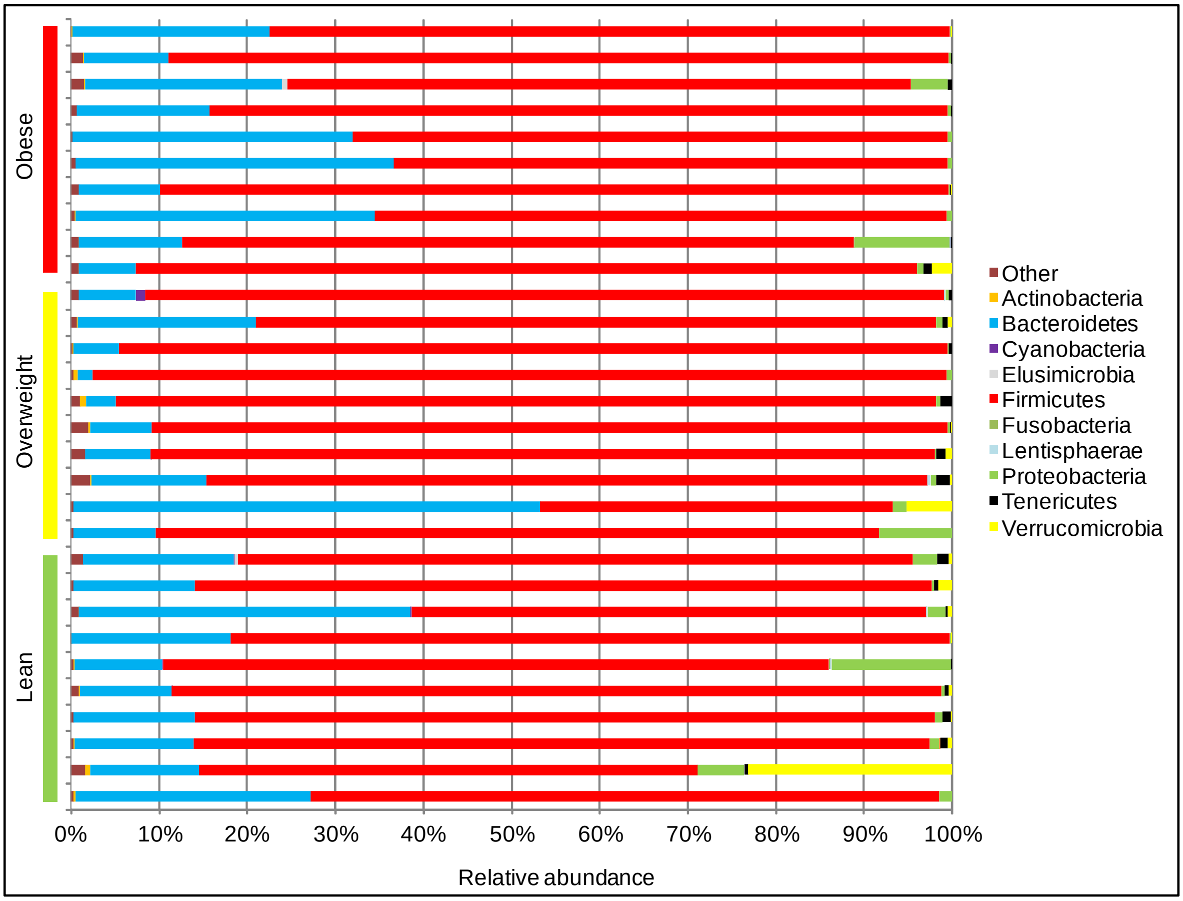

Supplementary Figure S4 - Correlations between the relative abundance of Firmicutes and Bacteroidetes with latitude

Grey points = data from Suzuki & Worobey 2014 (*Biology Letters* 10: 20131037); black points = data from this study (from left to right: Colombia, Japan, South Korea, USA, Europe [Spain, France, Denmark]). Pearson's *r* from correlation analyses; *P-value* from linear models.

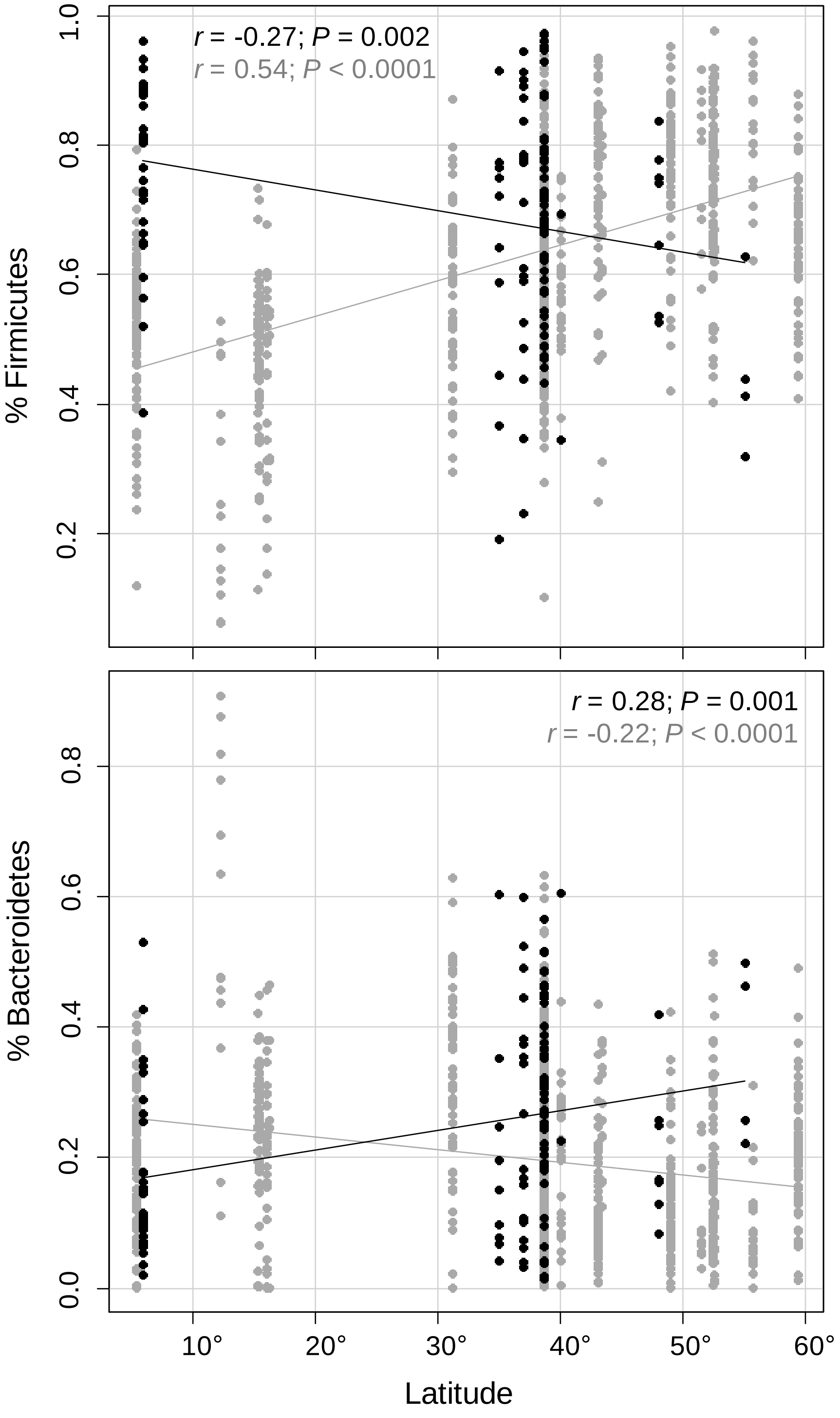

Supplement: Additional file 1: Table S1. — Some characteristics of the different datasets analyzed in this study. Figure S1 - Analysis pipeline. Figure S2 - Rarefaction curves in the different datasets. Figure S3 - Inter-individual variability of the gut microbiota among Colombians. Figure S4 - Correlations between the relative abundance of Firmicutes and Bacteroidetes with latitude. [file 12866_2014_311_MOESM1_ESM.pdf]
